# Acute Heat Exposure-Related Illness: A Unified Emergency Medicine Framework for Hot Baths, Hot Springs, and Saunas—A Narrative Review

**DOI:** 10.3390/jcm15051910

**Published:** 2026-03-03

**Authors:** Ryuto Yokoyama, Kenya Yarimizu, Tatsuya Hayasaka, Kento Sakaguchi, Masahiro Kuroki, Kiyotaka Soekawa, Tadahiro Kobayashi, Tsuneo Konta

**Affiliations:** 1 Department of Emergency and Critical Care Medicine, Yamagata University Hospital, Yamagata 990-9585, Japan; bealive32@yahoo.co.jp (K.S.); tad.kob12@gmail.com (T.K.); 2 Department of Anaesthesiology, Yamagata University Hospital, Yamagata 990-9585, Japan; yarimizu.kenya@gmail.com (K.Y.); hayasakatatsuya1101@gmail.com (T.H.); the_brilliant_greeeen@yahoo.co.jp (M.K.); k-soekawa@med.id.yamagata-u.ac.jp (K.S.); 3 Department of Public Health, Faculty of Medicine, Yamagata University, Yamagata 990-9585, Japan; kkonta@med.id.yamagata-u.ac.jp

**Keywords:** bath, bathing, hot spring, sauna, heat stroke, acute heat exposure, acute illness

## Abstract

Hot springs, hot-water bathing, and saunas are widely practiced forms of acute heat exposure and are often perceived as health-promoting. However, emergency clinicians frequently encounter patients in whom these exposures precipitate syncope, hypotension, drowning/aspiration, heat-related illness, and renal or electrolyte disturbances. This narrative review integrates these modalities within a unified “acute heat exposure” framework and summarizes pathophysiology and clinical implications from an emergency medicine perspective. We searched PubMed/MEDLINE from inception to January 2026 using controlled vocabulary and free-text terms related to heat stress, thermoregulation, hot-water immersion, sauna exposure, and acute clinical outcomes; evidence was synthesized qualitatively. Across modalities, acute heat exposure induces shared physiological responses—peripheral vasodilation, relative hypovolemia, circulatory stress, and internal heat storage—that can trigger diverse emergency presentations. We classify acute heat exposure–related illness into four domains: (1) cardiovascular events, including syncope, hypotension, and arrhythmic/ischemic complications in vulnerable individuals; (2) the heat-illness spectrum from exhaustion to heat stroke with organ dysfunction; (3) renal and electrolyte disturbances related to dehydration and hypoperfusion; and (4) neurological and traumatic complications, including falls, drowning, and aspiration. This framework may support risk stratification, evaluation, management, and prevention after hot spring, hot bath, or sauna use.

## 1. Introduction

Hot springs, bathing, and sauna bathing are widely practiced forms of acute heat exposure across cultures and regions and are generally considered beneficial behaviors for both physical and mental health [1,2]. In Japan, hot spring bathing has been linked to improved sleep quality [3], a lower prevalence of hypertension [4], and a reduced history of depression [5]. Additionally, epidemiological studies—primarily from Nordic countries—have suggested that sauna bathing is associated with reduced risks of cardiovascular mortality, all-cause mortality, and stroke, highlighting its potential as a health-promoting behavior [6,7,8].

However, in real-world emergency practice, clinicians routinely encounter patients in whom acute heat exposure serves as a clinically important trigger for adverse events [9,10]. These events are particularly common among older adults and patients with chronic diseases, in whom impaired physiological reserve against heat stress and circulatory stress is considered an important background factor [11,12,13]. Accordingly, a gap exists between the social perception of hot springs/bathing/sauna bathing as “healthy habits” and the reality in acute care, where they may act as “triggers for emergency conditions.” In this narrative review, we define acute heat exposure as physiological changes induced by relatively short periods of intense heat load (immersion and/or exposure to high ambient heat) related to hot springs/bathing/sauna bathing. Furthermore, we use the concept of acute heat exposure-related illness to encompass emergency-relevant conditions (e.g., heat syncope/exhaustion and heat stroke, cardiovascular events, neurological symptoms, kidney/electrolyte disturbances, trauma, drowning, and aspiration).

Moreover, heat transfer and hemodynamic effects differ between immersion-based exposure in hot springs/bathing and ambient acute heat exposure in saunas (including humidity conditions), which may alter the risks of accompanying events such as syncope, fall-related injuries, drowning, and aspiration.

Although existing guidelines are useful when acute heat exposure–related illness presents as typical heat-related conditions, acute manifestations may also involve the cardiovascular, respiratory, central nervous, and renal systems [14,15,16]. Current heat illness guidelines primarily focus on outdoor environmental exposure or exertional mechanisms, and integrated frameworks that explicitly incorporate exposure modalities specific to bathing/hot springs/saunas for differential diagnosis and triage remain limited.

Consequently, an implementable emergency medicine framework that integrates exposure modality and patient vulnerability (differential diagnosis, severity stratification, and early management) has not been sufficiently established.

This gap highlights the need for a clinically oriented review focusing on acute heat exposure. Prior studies and reviews have addressed acute heat exposure in a fragmented manner. For saunas, existing literature largely examines long-term cardiovascular outcomes or metabolic indices and tends to emphasize health benefits [17,18,19]. In contrast, adverse events related to hot springs and bathing are most frequently reported as accidental deaths and drowning, or are derived mainly from epidemiological studies and case series confined to specific regions (particularly Japan) [9,20,21,22,23]. This fragmentation generates at least three problems. First, studies focusing on chronic outcomes tend to select health-oriented populations, limiting applicability to risk estimation in acute care settings (healthy-user bias) [24]. Second, although hot springs, bathing, and saunas differ in exposure modalities and cultural contexts, they share core pathophysiological processes, including peripheral vasodilation, fluid loss, circulatory stress, and internal heat storage [25,26]; however, integrated clinical reviews remain scarce. Third, the central clinical question in emergency medicine—how acute heat exposure triggers emergency conditions and how clinicians should evaluate and respond in real-world practice—has not been adequately addressed. Consequently, emergency physicians and acute care clinicians lack a practical, cross-disease framework to understand acute heat exposure–related acute illness.

This narrative review aims to integrate hot springs, bathing, and sauna bathing under the shared framework of acute heat exposure and organize their clinical implications from an emergency medicine perspective. We specifically focus on shared physiological responses—peripheral vasodilation, relative reduction in effective circulating blood volume, circulatory stress, and internal heat storage—and describe how these can precipitate acute emergency conditions.

We organize the evidence as follows: (1) the spectrum of acute heat exposure–related illness (cardiovascular events, heat-related illness, kidney injury/electrolyte disturbances, and neurological/traumatic complications); (2) exposure modality–specific characteristics and emergency care considerations; and (3) high-risk populations. Additionally, we discuss the need for future research, including clarifying the relationship between internal heat storage and organ dysfunction, and emphasize the importance of quantitative assessment of acute heat exposure.

## 2. Materials and Methods

This narrative review was conducted to integrate an emergency medicine–oriented framework for acute heat exposure–related illnesses associated with hot-water tub bathing, hot springs, and saunas. We searched PubMed/MEDLINE from database inception to January 2026 using controlled vocabulary and free-text terms related to: (i) acute heat exposure and thermoregulation (e.g., heat stress, hyperthermia, heat-related illness, heat stroke, thermoregulation, and internal heat storage); (ii) bathing and hot-water immersion (e.g., bath, hot bath, hot-water immersion, hot spring, balneotherapy, and drowning); and (iii) sauna use (e.g., sauna and steam bath), combined with terms describing acute clinical outcomes (e.g., syncope, hypotension, arrhythmia, acute kidney injury (AKI), and electrolyte abnormalities). Study selection was performed in two stages as follows: (1) initial screening by title/abstract; and (2) full-text assessment. One author (R.Y.) conducted the screening; when uncertain, decisions were made in consultation with another author (K.Y.). Disagreements were resolved by discussion among co-authors.

Approximately 150 records were retrieved; after removing duplicates, we conducted primary screening, and among 108 full-text articles assessed, 97 were included. PubMed/MEDLINE was used as the primary database because it broadly covers peer-reviewed literature in emergency/clinical medicine and facilitates the identification of case reports, while enabling reproducible search strategies using MeSH terms. Although additional databases (e.g., Embase, Scopus, and Web of Science) were considered, this is a narrative review aimed at building an emergency medicine–oriented framework rather than a systematic review; therefore, PubMed/MEDLINE was chosen as the main database for stable retrieval of foundational clinical literature. To mitigate potential omissions inherent to a single database, we also screened reference lists of key papers and tracked citations in relevant reviews to identify additional important studies.

We included peer-reviewed articles published in English that provided clinically relevant evidence on: (a) physiological responses to acute heat exposure or hot-water immersion; (b) acute adverse events and emergency presentations related to bathing or sauna use (including drowning/aspiration and trauma); and (c) clinically relevant risk modifiers, including age, comorbidities, medications, and alcohol use. We prioritized higher levels of evidence when possible (systematic reviews, cohort studies, and population-based studies), while also including mechanistic/physiological studies to support clinical interpretation of emergency presentations. Reports that lacked relevance to acute illness or emergency care and focused only on long-term health benefits were not emphasized.

Because of substantial heterogeneity in exposure environments (water immersion vs. dry heat), study designs, and outcomes, we did not perform meta-analysis or formal risk-of-bias assessment using standardized tools. Instead, we qualitatively assessed methodological limitations (e.g., confounding and healthy-user bias, exposure misclassification, and incomplete outcome capture) and integrated these considerations into interpretation. Evidence was synthesized thematically according to: (1) core pathophysiological mechanisms (peripheral vasodilation, relative hypovolemia, circulatory stress, and internal heat storage); (2) spectrum of emergency conditions; (3) exposure modality–specific characteristics; (4) high-risk populations; and (5) implications for prevention strategies and future research.

## 3. Acute Heat Exposure as a Unified Pathophysiological Concept

### 3.1. Types of Acute Heat Exposure: Definition and Commonality

In this review, we focus on the following three representative forms of acute heat exposure: hot springs, bathing, and sauna bathing. Hot spring and tub bathing generally involve whole-body immersion, facilitating heat transfer from water to the body [27] and potentially causing increased skin temperature and hemodynamic changes via peripheral vasodilation even with short exposure durations [28]. Hot springs are commonly preferred at ~40–44 °C, whereas tub bathing is usually preferred at ~38–42 °C; however, temperature settings vary widely depending on individual preference and facility operations [2].

Regarding exposure duration, although 10–15 min bathing is recommended for safer use in Japan, longer immersion is frequently preferred in hot springs/bathing contexts [2]. In contrast, saunas primarily involve ambient acute heat exposure; physiological effects can vary by humidity (e.g., dry vs. wet/steam saunas). Typical sauna temperatures range from ~80 to 100 °C; conversely, heat load can increase depending on conditions [29,30]. Exposure duration also varies substantially based on individual preferences, facility practices, and cultural habits, and physiological responses may differ accordingly [31].

Although exposure modalities and cultural contexts differ, all three involve short-to-moderate exposure to high-temperature environments and share essential characteristics as acute heat exposure [1,2]. In high-temperature environments, the balance between heat production and dissipation shifts rapidly, imposing physiological strain [32]. From an emergency medicine perspective, it is critical that acute heat exposure simultaneously affects not only “body temperature” but also hemodynamics, fluid balance, and autonomic regulation [33,34,35]. Therefore, conceptualizing hot springs/bathing/sauna bathing as integrated forms of acute heat exposure may be useful for understanding and managing acute illnesses in emergency settings.

### 3.2. Core Physiological Responses

Acute heat exposure induces peripheral vasodilation to increase skin blood flow and promote heat dissipation. While essential for thermoregulation, this response can reduce venous return and decrease effective circulating blood volume [34]. Heat stress can impair endothelial homeostasis and promote increased vascular permeability and microcirculatory dysfunction through glycocalyx injury and the disruption of intercellular junctions [36,37]. Heat shock protein activation may interact with inflammatory mediators and cytokine responses, potentially amplifying or modulating inflammatory pathways [38,39]. Temperature-sensitive ion channels are also involved in controlling vascular tone and may contribute to variability in circulatory responses [40]. Additionally, acute heat exposure can compromise cardiac electrophysiological stability, manifesting as electrocardiogram (ECG) abnormalities and arrhythmias [41,42,43]. Furthermore, fluid loss due to sweating accelerates a relative hypovolemic state, and compensation may be insufficient in older adults and individuals using diuretics [44,45]. Consequently, even when measured body temperature elevation is mild, hemodynamic collapse can precede and present as emergency symptoms, including syncope and hypotension [33].

Heat storage reflects the net amount of heat accumulated in the body over time (heat gain > heat loss) and may not coincide with a single-point measurement of core temperature [46,47,48]. Particularly, immediately after exposure or after cooling has begun, substantial heat may remain stored in the skin/peripheral tissues (shell) or circulating blood even when core temperature is not markedly elevated; therefore, circulatory stress and symptoms may precede overt hyperthermia [49].

### 3.3. Conceptual Framework (Figure 1)

Hot baths, hot springs, and saunas are conceptualized as forms of acute heat exposure that elicit shared immediate physiological responses, including peripheral vasodilation, increased sweating, relative hypovolemia (reduced effective circulating volume), and reduced venous return. These responses collectively increase circulatory stress and, when heat gain exceeds heat loss, promote progressive internal heat storage, sympathetic activation, increased cardiac workload, and hemodynamic instability. As a result, acute heat exposure can precipitate a spectrum of emergency conditions, including cardiovascular events (syncope, hypotension, arrhythmia, and myocardial ischemia), heat-related illness (heat syncope, heat exhaustion, and heat stroke), renal and electrolyte disturbances (acute kidney injury and electrolyte disorders), and neurological/traumatic events (altered mental status, falls, and drowning). Modifying factors—such as older age, comorbidities, medications, and alcohol intake—may amplify or mitigate these pathways and influence clinical outcomes.

**Figure 1:**
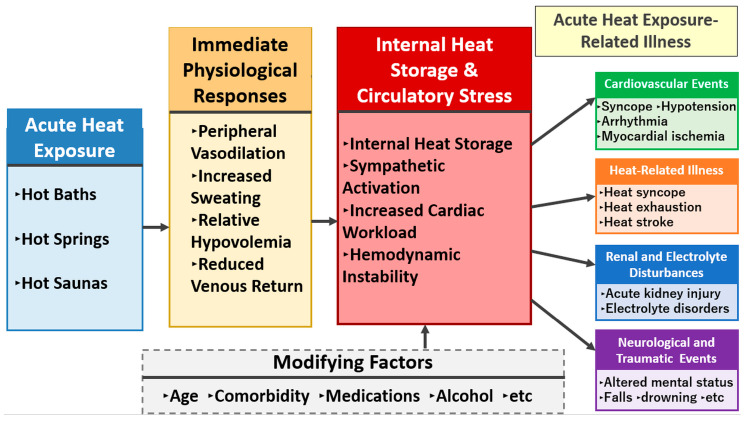
Conceptual framework linking acute heat exposure to heat exposure–related acute illness.

Conceptualizing exposure as a process involving internal heat storage and circulatory stress, rather than focusing solely on “temperature”, is crucial for understanding adverse events related to acute heat exposure [38,44,50,51]. Acute heat exposure increases circulatory stress through peripheral vasodilation and fluid loss, and internal heat storage progresses when heat dissipation fails to match heat gain. The cumulative burden of internal heat storage and circulatory stress can lead to acute illnesses—including circulatory collapse, altered mental status, trauma, and organ injury—via impaired organ perfusion and thermoregulatory failure.

### 3.4. Internal Heat Storage and Circulatory Stress

When acute heat exposure persists or heat dissipation is insufficient, heat production exceeds heat loss, leading to progressive internal heat storage [38,44,50,51]. Internal heat storage is not synonymous with a single measured body temperature value; however, it is a conceptual component closely related to circulatory stress and organ injury. As internal heat storage increases, hemodynamic stress may appear (tachycardia, changes in cardiac output, and blood pressure instability), and in patients with limited cardiovascular reserve, arrhythmias and ischemic events may be triggered [25,52].

Clinically, internal heat storage may not coincide with a single measured body temperature. In emergency settings, exposure intensity (temperature), duration, and time to cooling are often insufficiently assessed; consequently, clinicians may overlook ongoing circulatory failure or organ injury in patients whose measured temperature is “not very high” [51].

### 3.5. Individual Susceptibility and Background Factors as Modifiers

Responses to acute heat exposure vary substantially depending on modifiers, including age, comorbidities, medications, and alcohol use. For example, older adults have impaired thermoregulatory and circulatory regulation; patients with diabetes, chronic kidney disease, or cardiovascular disease may have difficulty maintaining fluid and circulatory homeostasis [44,53]. Diuretics and β-blockers may limit compensatory mechanisms and increase the risk of adverse events [54,55,56]. These background factors can drive divergent clinical trajectories even under similar exposures and directly inform risk stratification in emergency care.

## 4. Acute Heat Exposure–Related Illness

### 4.1. Cardiovascular and Circulatory Events

Syncope and hypotension represent common emergency conditions associated with acute heat exposure [33]. Peripheral vasodilation and relative hypovolemia can cause transient cerebral hypoperfusion, with symptoms frequently occurring during postural change (e.g., standing up or exiting the bathtub) [34]. In patients with cardiovascular disease, increased circulatory stress and autonomic fluctuations may precipitate arrhythmias and ischemic heart disease events [50,52]. Therefore, detailed history-taking regarding recent acute heat exposure is essential in emergency settings. To aid in understanding cardiovascular and circulatory events, we reviewed relevant literature and summarized the findings in Table 1.

### 4.2. Heat-Related Illness Continuum

Although hot springs/bathing/sauna bathing differ from outdoor environmental acute heat exposure, they can be interpreted within the continuum of heat-related illness, ranging from heat exhaustion to heat stroke.

#### 4.2.1. Pathophysiology

As heat load increases, thermoregulation relies on elevated skin blood flow and sweating for heat dissipation [11,63]. However, when relative reductions in circulating blood volume due to peripheral vasodilation, dehydration from sweating/diuresis, and circulatory redistribution during immersion are superimposed, compensation by cardiac output may become insufficient, leading to circulatory collapse [11,64]. With prolonged exposure and delayed/inadequate cooling, internal heat storage progresses and may evolve into severe heat stroke with altered mental status and multiple organ failure [65,66,67].

#### 4.2.2. Clinical Presentations (Typical Signs/Symptoms) [65,66]

Heat syncope/heat exhaustion (mild): This presents with symptoms including fatigue, dizziness, nausea, headache, muscle cramps, and lightheadedness. Flushed skin and sweating are frequently preserved. Hypotension and tachycardia may be present; signs of dehydration (thirst and dry mucosa) may occur. Heat exhaustion (moderate): This is characterized by weakness, ataxia, repeated vomiting, and difficulty walking. With progressive circulatory failure, decreased consciousness may develop. Heat stroke (severe): This is marked by high body temperature with central nervous system dysfunction (delirium, seizures, and coma) and organ injury; complications may include rhabdomyolysis, liver injury, kidney injury, coagulopathy, and shock.

#### 4.2.3. Outcomes

Outcomes are strongly determined by (1) the presence of central nervous system symptoms, (2) severity of circulatory failure, and (3) progression of organ injury (the kidneys, liver, coagulation systems, and muscles) [68,69]. Heat exhaustion frequently improves with appropriate cooling and fluid resuscitation, whereas heat stroke may have worse outcomes when aggressive early cooling is delayed [66,67]. In high-risk groups—including patients with core temperature ≥ 40 °C or older adults—heat-related complications are associated with extremely poor outcomes, with one study reporting a 1-year survival rate as low as 18% [70]. Therefore, emergency evaluation should integrate not only temperature but also exposure duration, time to cooling, hemodynamics, and mental status. When severe illness is suspected, active cooling and circulatory support should be initiated early [66,67].

### 4.3. Renal and Electrolyte Disturbances

#### 4.3.1. Pathophysiology

Sweating and dehydration can precipitate AKI and electrolyte abnormalities [71]. Kidney injury may arise from overlapping mechanisms, including: (1) reduced effective circulating blood volume due to dehydration and peripheral vasodilation (renal hypoperfusion); (2) renal ischemia associated with circulatory collapse and inflammatory responses; and (3) myoglobinuria due to rhabdomyolysis [72,73,74]. In older adults and patients with chronic kidney disease, even mild fluid loss may cause clinically significant renal dysfunction [75]. In severe heat stroke, endothelial injury, coagulopathy, and amplified inflammation can accelerate kidney injury, potentially leading to oliguria and metabolic acidosis [74,75].

#### 4.3.2. Typical Clinical/Laboratory Findings

AKI [71,72]: This is characterized by oliguria, concentrated urine, and elevated serum creatinine, and may be accompanied by electrolyte and acid–base disturbances.

Rhabdomyolysis [73]: This presents with myalgia, weakness, and dark urine (myoglobinuria); elevated creatine kinase is a key marker, and it may complicate AKI.

Electrolyte abnormalities can include [76,77]: hyper-/hyponatremia, which may cause altered mental status and seizures; hyper-/hypokalemia, which can cause arrhythmias and weakness; and hyper-/hypocalcemia, which may cause arrhythmias, neuromuscular symptoms, and neurological manifestations.

#### 4.3.3. Outcomes and Clinical Implications

Kidney injury and electrolyte abnormalities may influence outcomes through arrhythmias, seizures, altered mental status, and circulatory failure [74,78]. Particularly, AKI with rhabdomyolysis, severe Na/K derangements, and progressive acidosis are warning signs of severe disease and frequently warrant observation or inpatient management [73,74]. Accordingly, in patients suspected of acute heat exposure–related illness, evaluating kidney function, checking electrolyte levels, and performing ECG monitoring are important.

### 4.4. Neurological and Traumatic Events

Peripheral vasodilation and hypotension associated with acute heat exposure can reduce cerebral perfusion, causing altered mental status, syncope, and dizziness [33,34]. Severe disease may present with hyperthermia-related central nervous system injury (delirium, seizures, and coma) [65,66]. Moreover, when altered mental status or syncope occurs during bathing, it can directly lead to aspiration or drowning, and many patients present to the emergency department with respiratory failure [9]. Falls associated with syncope or altered consciousness also occur and can result in head or torso trauma [79,80]. In older adults and those taking anticoagulants, even minor falls may be severe; therefore, emergency evaluation should systematically include trauma assessment in addition to neurological evaluation [81,82]. This syndrome should be understood as a complex condition where “primary acute heat exposure–related impairment” is compounded by secondary complications, including aspiration, drowning, and trauma.

## 5. Exposure-Specific Characteristics (Table 2)

Table 2 summarizes exposure characteristics, core physiological effects, typical acute emergency conditions, and key considerations for emergency physicians across acute heat exposure modalities, including hot-water tub bathing/hot springs and sauna bathing. Although the modalities differ in exposure duration, temperature, and hydrostatic pressure, common pathophysiological processes—including peripheral vasodilation, relative hypovolemia, circulatory stress, and internal heat storage—underlie many acute clinical presentations. From an emergency medicine perspective, outcomes are frequently determined more by the interaction between exposure intensity/duration and patient vulnerability than by the exposure type.

**Table 2:** Exposure-specific emergency conditions and clinical features. Summary of exposure modality (immersion vs. ambient heat), typical exposure parameters, core physiological effects, and acute emergency conditions relevant to hot baths/hot springs and saunas.

Setting	Modality and Typical Parameters	Physiological Effects	Acute Conditions	ED Considerations	Key References
Hot baths/hot springs	Immersion in hot water; hydrostatic pressure. Typical water temperature ~38–42 °C (home baths) and ~40–44 °C (hot springs); typical immersion time ~10–15 min.	Peripheral vasodilation; reduced venous return; BP lability; internal heat storage; and dehydration with prolonged exposure.	Syncope, hypotension, drowning, aspiration pneumonia, heat illness, and arrhythmia or ischemia (susceptible).	Ask about duration, temperature, alcohol intake, and post-bath standing. Solitary bathing increases delayed-rescue risk (older adults).	[2,9,10]
Saunas	Ambient heat without immersion and intense sweating. Dry sauna typically ~80–100 °C with low humidity (~10–20%).	Rapid fluid loss; relative hypovolemia; vasodilation; cardiovascular strain; and internal heat storage.	Syncope, hypotension, arrhythmia, ischemic events, heat exhaustion/heat stroke, and secondary trauma.	Assess hydration and CV history; caution with rapid standing and contrast therapy (cold plunge/shower). Consider alcohol or drugs and medications.	[1,6,7,16,29,30]
Shared features (acute heat exposure)	High thermal load; higher intensity and longer duration increase risk, especially in vulnerable individuals.	Vasodilation + fluid loss -> circulatory stress; internal heat storage; and organ dysfunction if severe.	Heat illness, AKI, electrolyte disorders, neurologic impairment, and secondary trauma.	Severity is frequently driven by intensity and duration plus patient vulnerability rather than setting alone; early recognition and cooling and rehydration are key.	[16,65,66]

Abbreviations: ED, emergency department; BP, blood pressure; CV, cardiovascular; AKI, acute kidney injury.

### 5.1. Hot Baths and Hot Springs

Hot springs and hot-water tub bathing involve sustained whole-body immersion [2]. Hydrostatic pressure can alter venous return, and postural change before and after bathing may induce marked blood pressure fluctuations (so-called “heat shock”), leading to hemodynamic instability [57,83]. In Japan, high-temperature and prolonged bathing are culturally accepted, and older adults frequently bathe alone [84]. These circumstances may delay intervention when syncope or altered mental status occurs, increasing the risk of progression to drowning, aspiration, and severe respiratory failure [9,10,85]. Therefore, behavioral factors—including bathing duration, water temperature, and alcohol intake—should be recognized as contributors to adverse events.

### 5.2. Saunas

Sauna bathing is characterized by short exposure to high ambient temperatures and can induce rapid sweating and fluid loss [30]. Given that immersion is absent, hydrostatic pressure effects are limited; however, dehydration and peripheral vasodilation may rapidly progress to a relative hypovolemic state and increase cardiac workload [34]. In individuals with cardiovascular disease, increased circulatory stress may precipitate arrhythmias and ischemic events [50,52]. Recently, increased sauna use and the popularity of repeated sauna–cold water immersion cycles have raised concerns that rapid temperature shifts may impose substantial hemodynamic stress [29], warranting further investigation.

### 5.3. Differences Across Exposure Modalities and Shared Features

Although hot springs/bathing/saunas differ in exposure duration, temperature, and hydrostatic pressure, they share common pathophysiological features, including peripheral vasodilation, fluid loss, circulatory stress, and internal heat storage [12,34,50]. From an emergency medicine perspective, acute outcomes may be more strongly governed by the interaction between exposure intensity/duration and individual vulnerability than by the exposure modality. Therefore, these exposures should be evaluated comprehensively as acute heat exposure rather than as isolated behaviors.

## 6. High-Risk Populations (Table 3)

Table 3 outlines populations more likely to deteriorate or develop complex courses after acute heat exposure, along with underlying pathophysiological vulnerabilities and typical complications. Older adults, patients with cardiovascular or chronic kidney disease, and individuals using medications that blunt compensatory responses may develop marked instability even with relatively mild exposures. Recognizing these high-risk features is crucial for early risk stratification and appropriate management in emergency settings.

**Table 3:** High-risk populations and clinical considerations in acute heat exposure. Highlights patient-level vulnerability factors that amplify circulatory stress, dehydration risk, or impaired thermoregulation.

High-Risk Population/Factor	Pathophysiological Vulnerability	Typical Acute Complications	Key Clinical Implications	Key References
Older adults	Impaired thermoregulation, reduced CV reserve, blunted thirst, and solitary bathing are more common.	Syncope, hypotension, heat stroke, drowning, and aspiration.	Even mild exposure may destabilize. Get detailed exposure history and rescue context, and intervene early.	[2,21,22]
Cardiovascular disease	Limited compensation for vasodilation and tachycardia, and arrhythmia susceptibility.	Arrhythmia, myocardial ischemia, collapse, and syncope.	Events may occur without marked hyperthermia; consider ECG and biomarkers when indicated.	[6,7,16]
Chronic kidney disease	Reduced ability to maintain volume and electrolyte balance.	AKI, electrolyte disorders, and rhabdomyolysis (severe).	Small dehydration can cause major dysfunction; check renal function and electrolytes early.	[16,71,72]
Diuretic use	Baseline volume depletion; impaired compensatory response.	Hypotension, AKI, syncope, and electrolyte derangements.	Medication history is key; correct volume/electrolytes and adjust contributing agents.	[16]
Diabetes with autonomic dysfunction	Impaired vasomotor/sweating responses, dehydration risk, and reduced symptom awareness.	Hypotension, altered mental status, and heat illness.	Autonomic impairment may mask early signs; lower threshold for monitoring and active cooling.	[16]
Alcohol consumption	Vasodilation + diuresis, impaired judgment, prolonged exposure, and delayed rescue.	Syncope, drowning, aspiration, and trauma.	Screen intake, and anticipate delayed presentation and coexisting hypoglycemia or trauma.	[2,9]
Drug abuse (including stimulants and psychoactive substances)	Impaired judgment, reduced heat perception, higher metabolic heat (stimulants), dehydration, and pro-arrhythmic effects.	Severe heat illness/heat stroke, arrhythmia, agitation, rhabdomyolysis, and trauma.	Include substance use in triage; consider toxidromes/co-ingestions; and monitor temperature, ECG, and CK.	[16,54]
History of heat-related illness	Reduced tolerance to thermal stress and possible residual organ vulnerability.	Recurrent heat illness; dehydration-related complications.	Prior episodes suggest heightened risk; reinforce prevention and early ED evaluation for recurrence.	[16]

Abbreviations: ED, emergency department; ECG, electrocardiogram; CV, cardiovascular; AKI, acute kidney injury; CK, creatine kinase.

Adverse events related to acute heat exposure tend to be more severe in specific populations. The most important high-risk group is older adults; impaired thermoregulation and circulatory regulation can lead to syncope and heat-related illness even with mild exposure [44,78]. Patients with cardiovascular disease have reduced cardiovascular reserve and higher risks of arrhythmias and ischemic events [34,52]. Patients with chronic kidney disease have impaired fluid/electrolyte regulation and are susceptible to dehydration and AKI [71]. Additionally, diuretic use, β-blocker use, diabetic autonomic dysfunction, and alcohol use may increase risk [34,86]. A prior history of heat illness suggests elevated recurrence risk and warrants caution [12,51].

## 7. Preventive Strategies for Acute Heat Exposure–Related Acute Illness

### 7.1. General Principles Applicable to All Acute Heat Exposure Settings

The most important shared preventive principle for hot springs/bathing/saunas is to avoid excessive progression of internal heat storage and circulatory stress [13,34,50]. First, limiting exposure duration is essential. Longer exposure increases the imbalance between heat gain and loss, accelerating internal heat storage [87]. In older adults and those with comorbidities, hemodynamic compensation may fail even with short exposure; therefore, conservative time limits are warranted [44,57]. Second, appropriate hydration is critical. Fluid loss from sweating increases circulatory stress, whereas excessive intake of free water alone may cause hyponatremia [71]. In high-risk individuals, consuming beverages containing electrolytes and avoiding excessive free-water intake are advisable. Third, acute heat exposure after alcohol intake should be avoided. Alcohol can increase adverse event risk through peripheral vasodilation, impaired judgment, and thermoregulatory dysfunction [88].

### 7.2. Preventive Strategies for Hot Baths and Hot Springs

In hot springs and bathing, because whole-body immersion and postural change strongly affect hemodynamics, prevention should focus primarily on circulatory collapse and drowning risk [89]. First, optimizing water temperature is important. High-temperature bathing increases the risk of hemodynamic failure; therefore, very hot (e.g., ≥41 °C) and prolonged bathing should be avoided [90]. Second, abrupt postural changes should be avoided. Standing up and exiting the bathtub can precipitate syncope through peripheral vasodilation and reduced venous return; gradual, slow movements should be encouraged [58,84]. Third, avoiding solitary bathing is an important safety measure. In older adults and high-risk individuals, altered mental status during bathing may delay intervention and increase drowning/aspiration risk [9].

### 7.3. Preventive Strategies for Sauna Use

In saunas, rapid sweating and fluid loss can occur within short periods; therefore, managing dehydration and circulatory stress is key [30]. First, continuous or excessively repeated sessions should be avoided [1]. Particularly, sauna–cold water immersion repetition may impose substantial hemodynamic stress due to rapid temperature shifts [87].

Second, hydration and electrolyte replenishment before and after sauna use are important. For individuals with cardiovascular disease or chronic kidney disease, clinicians should emphasize the risks of dehydration-related circulatory collapse and kidney function deterioration [26,38]. Third, sauna use should be avoided during acute illness or exacerbations of underlying disease. Sauna exposure during fever, infection, or dehydration may rapidly amplify internal heat storage and circulatory stress [12,38].

### 7.4. High-Risk Populations and Tailored Preventive Advice

As summarized in Section 6, older adults, patients with cardiovascular disease, those with chronic kidney disease, and individuals using diuretics or β-blockers have higher risks of adverse events from acute heat exposure. In addition to general preventive measures, tailored advice is needed: stricter limits on frequency and duration, supervision by family/caregivers, and immediate cessation with any symptom development.

### 7.5. The Role of Emergency Medicine in Prevention and Recurrence Reduction

Emergency medicine plays a crucial role not only in acute management but also as an intervention point for secondary and primary prevention. For cases of acute heat exposure–related illness that correspond to typical heat illness presentations, the Wilderness Medical Society clinical practice guidelines provide practical recommendations for prevention and treatment in emergency and out-of-hospital settings [16]. For severe cases, including cardiac arrest, the American Heart Association and European Resuscitation Council special circumstances guidelines provide key points for resuscitation and initial management in hyperthermia/heat stroke and other special situations [14,15]. Emergency physicians should apply these principles while managing acute heat exposure–related illness as a complex syndrome. Providing specific preventive counseling based on exposure modality, behavioral factors, and individual risks to patients presenting with acute heat exposure–related illness may help reduce future severe events [65]. Reframing acute heat exposure as an “adjustable medical risk factor,” rather than an “inevitable daily behavior,” is increasingly important for both emergency medicine and public health [91].

## 8. Limitations of Current Evidence

Current evidence on acute heat exposure has important limitations. First, the existing literature largely relies on observational studies or case series, limiting rigorous causal inference between exposure and outcomes. Sauna epidemiology is particularly susceptible to healthy-user bias, requiring caution when extrapolating to risk estimation in acute care settings. Second, quantitative characterization of exposure (temperature, duration, frequency, and behavioral patterns) is frequently inadequate, leading to substantial heterogeneity across studies. Third, relying on a single body temperature measurement may fail to capture peak heat load during exposure or internal heat storage. Furthermore, mild cases and individuals who do not seek medical care are difficult to capture, and reporting bias may influence observed patterns. These limitations hinder accurate characterization of the true risk profile of acute heat exposure.

## 9. Future Research Directions

### 9.1. Need for Quantitative Assessment

To deepen understanding of acute heat exposure–related illness, establishing quantitative and integrated assessment approaches is essential [92]. Rather than relying on a single measured temperature value, assessing internal heat storage based on the balance between heat gain and loss is crucial [93]. Heat balance models and continuous temperature monitoring can quantify heat stress while accounting for exposure modality and inter-individual differences, providing a basis for more precise investigation of its relationship with organ injury and severity indices [38,87].

### 9.2. Implications for Future Studies

Multicenter prospective studies are needed to systematically collect and evaluate emergency-transported cases related to hot springs/bathing, including exposure conditions, comorbidities, initial management, and clinical outcomes [37]. Such studies can provide implementable evidence reflecting regional and cultural contexts and inform prevention strategies. Additionally, research examining links between acute heat exposure and multiple organ failure from the perspective of hemodynamics and organ perfusion, and clarifying pathophysiological progression mediated by internal heat storage, is also important [50,70]. Finally, interdisciplinary research spanning emergency and environmental medicine is needed to position acute heat exposure not as an “accidental event,” but as a “predictable, assessable, and modifiable medical risk factor.”

## 10. Conclusions

In this narrative review, we organized the unified concepts of acute heat exposure and related illness across hot springs, bathing, and sauna bathing in a manner that is practical for emergency medicine. These environments may differ in how acute heat exposure–related illness manifests due to modality-specific characteristics. Nevertheless, common pathophysiological mechanisms include circulatory collapse originating from peripheral vasodilation and a relative reduction in effective circulating blood volume, dehydration and electrolyte disturbances, and—particularly in severe cases—central nervous system dysfunction and organ injury. Clinically, conceptualizing these conditions along a continuum from heat syncope/exhaustion to heat stroke is useful.

Practical implications for emergency care include rapidly stratifying patients with suspected acute heat exposure into: (1) severe illness requiring immediate cooling and resuscitation (heat stroke, altered mental status, and circulatory failure); (2) moderate illness requiring evaluation of complications including arrhythmias and renal/electrolyte disturbances; and (3) mild illness suitable for short observation and discharge with preventive counseling. When severe illness is suspected, early initiation of active cooling is critical, alongside evaluation for ECG abnormalities, electrolyte disturbances, kidney injury, rhabdomyolysis, and concomitant trauma/drowning. Future studies should quantify exposure modalities and clinical outcomes, clarify mechanisms and risk prediction for acute heat exposure–related illness, and test preventive interventions accounting for facility and cultural differences—thereby enhancing emergency care and informing effective prevention strategies.

## Figures and Tables

**Table 1:** Cardiovascular and circulatory events associated with hot-water immersion, hot spring bathing, and sauna exposure. Selected studies with clinically interpretable cardiovascular/circulatory endpoints and exposure characterization.

Study	Exposure Modality (Typical Conditions)	Design/Setting	Sample Size (*n*)	Population	Cardiovascular/Circulatory Event(s) Assessed	Main Outcomes/Key Findings
[6]	Sauna frequency (1/wk vs. 2–3/wk vs. 4–7/wk) and duration	Prospective cohort (Kuopio Ischemic Heart Disease study; baseline 1984–1989)	2315 men; median follow-up 20.7 years	Finnish men aged 42–60 years	Fatal outcomes: sudden cardiac death, fatal CHD, fatal CVD, and all-cause mortality	Higher sauna frequency associated with lower fatal CVD outcomes (adjusted HR for SCD 0.37 for 4–7/wk vs. 1/wk); longer sessions (>19 min) also associated with lower SCD risk vs. <11 min.
[10]	Home bathing/hot-tub related events (Japan; winter season emphasized)	Prospective cross-sectional observational surveillance (Tokyo, Saga, Yamagata; October 2012–March 2013)	4593 events (1528 cardiac arrests; 935 “survivors in need of help”; 1553 acute illnesses; 577 injuries)	EMS-activated bath-related events; predominantly older adults	Bath-related cardiac arrest; syncope/altered consciousness; acute illness; drowning mechanism	A large number of bath-related cardiac arrests; ACS/stroke were infrequently diagnosed among survivors; 30% of survivors had T > 38 °C; findings suggest heat illness during immersion contributes to loss of consciousness and drowning.
[57]	Real-world hot-tub bathing; typical water ~41 °C; deep tub often to neck (Japan)	Cross-sectional cohort survey with 24 h ambulatory BP/PR + skin temperature (January 2016–April 2019)	1479 participants (1169 hot bath immersion; 310 shower/warm bath)	Community-dwelling older adults (median age 68; 40–90 years; 548 men/931 women)	Hemodynamic changes during/around bathing (SBP, PR, and double product); syncope risk framework	Higher proximal skin temperature during bathing associated with higher SBP and PR; colder pre-bath indoor temperature associated with higher maximal skin temperature during bathing, suggesting potential for excessive hemodynamic changes.
[58]	Hot bath immersion (40 °C; shoulder level; 10 min)	Physiologic experiment with HRV and hemodynamics	18 (9 elderly; 9 young)	Elderly men (mean age ~75 years) vs. healthy young men (mean age ~27 years)	Hemodynamic instability (BP/HR changes); autonomic imbalance; myocardial oxygen demand (double product)	The elderly showed reduced autonomic responsiveness; transient imbalance may predispose to hypotension/bradycardia and syncope. The double product increased immediately after immersion, suggesting increased myocardial oxygen demand early in exposure.
[59]	Sauna bathing (monitored session; protocol-defined conditions)	Within-subject comparison: rest vs. exercise vs. sauna; ECG + BP + Tc-99m sestamibi scintigraphy	16 patients with CAD (15 with perfusion defect scoring)	Stable coronary artery disease	Myocardial ischemia (scintigraphic perfusion defects); arrhythmias/ECG changes	Sauna increased HR and reduced SBP; no arrhythmias/ECG changes; however, scintigraphy showed worse perfusion defect scores vs. rest; sauna-induced ischemia correlated with exercise-induced ischemia.
[60]	Warm-water bath (41 °C, 10 min) or sauna bath (60 °C, 15 min)	Invasive hemodynamic study with Swan–Ganz catheter; measurements before/during/after	34 patients with CHF (NYHA II–IV; mean EF 25%)	Chronic congestive heart failure	Hemodynamic response (SVR, cardiac index, filling pressures), and BP/HR; safety	Cardiac/stroke indexes increased, and SVR decreased during/after both exposures; filling pressures changed during exposure but decreased below baseline after; overall hemodynamics improved with careful thermal vasodilation.
[61]	Hot spring bathing (40 °C, 10 min daily for 2 weeks)	Randomized controlled trial (balneotherapy vs. daily shower control)	32 patients with systolic CHF (NYHA II–III)	Chronic heart failure	Cardiac function and biomarkers (EF and BNP), and inflammatory markers	After 2 weeks, symptoms improved; EF increased, and BNP decreased; hsCRP, TNF-α and IL-6 decreased in the balneotherapy group.
[62]	Tub bathing frequency (0–2/week to almost daily/every day)	Prospective cohort (Japan; long-term follow-up)	30,076 participants; 538,373 person-years; 2097 incident CVD events	Middle-aged Japanese adults	Incident CVD (CHD, stroke subtypes) and sudden cardiac death	Higher tub bathing frequency associated with lower incident total CVD and stroke risk (e.g., adjusted HR 0.72 for almost daily vs. 0–2/week for total CVD).

**EMS**, emergency medical services; **ACS**, acute coronary syndrome; **BP/PR**, blood pressure/pulse rate; **SBP**, systolic blood pressure; **HR**, heart rate; **HRV**, heart rate variability; **CAD**, coronary artery disease; **ECG**, electrocardiogram; **CHF**, congestive heart failure; **NYHA**, New York Heart Association (functional classification); **EF**, ejection fraction; **SVR**, systemic vascular resistance; **BNP**, B-type natriuretic peptide; **hsCRP**, high-sensitivity C-reactive protein; **TNF-α**, tumor necrosis factor-alpha; **IL-6**, interleukin-6; **CVD**, cardiovascular disease; **CHD**, coronary heart disease; **SCD**, sudden cardiac death.

## Data Availability

Not applicable. No new data were created or analyzed in this study.
